# Advantages and Challenges of A Village Doctor-Based Cognitive Behavioral Therapy for Late-Life Depression in Rural China: A Qualitative Study

**DOI:** 10.1371/journal.pone.0137555

**Published:** 2015-09-15

**Authors:** Xinfeng Tang, Fahui Yang, Tan Tang, Xuemei Yang, Weijun Zhang, Xiaohua Wang, Li Ji, Yun Xiao, Kun Ma, Ying Wang, Xianglei Kong, Jianping Wang, Jun Liu, Qian Xu, Donghua Tian, Zhiyong Qu

**Affiliations:** 1 Center for Behavioral Health, Beijing Normal University, Beijing, China; 2 School of Social Development and Public Policy, Beijing Normal University, Beijing, China; 3 Faculty of Humanities and Social Science, City University of Macau, Macau, China; 4 Department of Social Work, The Chinese University of Hong Kong, Hong Kong, China; 5 Mental Health Education Center of College Student, Sichuan Normal University, Chengdu, China; 6 China Institute of Health, Beijing Normal University, Beijing, China; 7 Mianzhu Ankang Hospital, Deyang, China; 8 Mianzhu Maternal and Child Health Hospital, Mianzhu County, Sichuan Province, China; 9 School of Psychology, Beijing Normal University, Beijing, China; 10 Department of Clinical Psychology, Capital Medical University, Beijing, China; 11 Department of Psychiatry and Clinical Psychology, Beijing Anding Hospital, Capital Medical University, Beijing, China; 12 Center for Mental Health, Southwestern University Of Finance And Economics, Chengdu, China; Institute of Automation, Chinese Academy of Sciences, CHINA

## Abstract

**Background:**

The delivery of mental health services in rural China has been notably limited due to lack of qualified mental health professionals among other impeding factors. A village doctor-based cognitive behavioral therapy intervention may be one way of improving accessibility. The purpose of this study was to explore the advantages and challenges of implementing this intervention, as delivered by trained village doctors, to treat late-life depression in rural China.

**Methods:**

We conducted one focus group discussion with 10 village doctors, 10 individual interviews with each of the village doctors, and individual interviews with 19 older adults. The topic guides were advantages and challenges of the intervention program from the perspective of the village doctors and older adults. Interviews were audio-recorded, transcribed, coded using NVivo 8, and analyzed using thematic analysis.

**Results:**

The village doctors stressed the importance of role-playing and using instructive manuals in the training. Proper supervision was also a key component of the program. The benefits received from the intervention for the village doctors and the elders were positive such that both the doctors and the older adults were willing to implement/receive this intervention. Cultural and political factors (*renqing* and perceived policy consideration) facilitated the elders’ access to mental health services. Challenges included a lack of real therapy (in contrast to role-playing) demonstrated in the training and lack of a step-by-step manual based on different types of problems encountered. Other impediments to the successful implementation of the intervention included the time constraints of village doctors and the presence of other people when conducting the intervention.

**Conclusions:**

The present study has demonstrated that the intervention program is likely to be an acceptable geriatric depression intervention in rural China if several challenges are appropriately addressed.

## Introduction

Depression in the elderly is a major public health concern that is associated with considerable morbidity [1–3] and increased suicide risk [4,5]. The self-reported prevalence rate of depression of Chinese older adults aged 50 and older is 0.3% and is considerably higher when a symptom-reporting and diagnostic algorithm is used (2%) according to a nationwide population-based older adults’ health and well-being survey in China [6]. Though the depression rate among older adults is considerably lower in China than among Western older populations, the large number of old people in China—more than 200 million elderly people lived in China in 2013 [6]—makes depression a serious social problem.

Pharmacotherapy and psychotherapy are currently recommended for treatment of depression in adults of any age [7]. Among the four main psychotherapeutic treatment groups (psychodynamic therapy, interpersonal therapy, supportive counseling and cognitive behavioral therapy), cognitive behavioral therapy (CBT) is the most researched form of psychotherapy [8,9]. Though CBT has been determined to be effective for the treatment of depression in older adults [7,9], most people living in low- and middle-income countries (LMIC), including China, do not have access to these treatments, especially in rural areas [10].

Several important reasons are attributable to this treatment gap for depression among rural elders: firstly, community mental health settings primarily provide services for persons with severe mental illness. The treatment of depression is not incorporated into services, although depression does, in fact, have a high prevalence rate [11]. Secondly, the diagnosis, prevention, and treatment of mental illness in rural older adults are largely ignored, which results in this population’s having little access to mental health services. Thirdly, few rural health workers (village doctors) have received mental health training. The lack of skilled professionals is a key limiting factor in implementing mental health services in low-resource settings. [10,12,13].

Designing a method of service delivery that fits in with the existing health system may be successful in reducing the treatment gap [14]. In rural China, primary health care and public health services (especially for older adults) are delivered by 1.303 million village doctors, averaging approximately 2.05 village doctors in each village (National Health and Family Planning Commission of the PRC, 2013). Therefore, we developed a village doctor-based psychological intervention, designated Brief Comprehensive Cognitive Behavioral Therapy (BC-CBT). BC-CBT, based on the principles of CBT, is designed specifically for village doctors working with older people with depression. It is our hope that this intervention prove to be an acceptable, feasible and effective treatment for depression in under-resourced settings. We conducted a qualitative study to explore the advantages and challenges of delivering and receiving such an intervention from the perspective of the trained staff and service users.

## Methods

### Ethics Statement

This study was approved by the Review Board of the School of Social Development and Public Policy in Beijing Normal University.

### Training and Intervention

#### Training manual and therapist manual

BC-CBT was adapted specifically for village doctors (VDs) and older adults in rural areas. The intervention was based on cognitive behavioral therapy, with the emphasis on “psychotherapy”, rather than purely psychoeducation or behavioral techniques. Cognitive behavioral therapy states that depressed individuals have stable cognitive schemas (also referred to as underlying assumptions or core beliefs) that would predispose people toward negative interpretations of life events (i.e., cognitive distortions or automatic thoughts), which, in turn, lead the depressed person to engage in depressive behavior. CBT for depression includes interventions that focus on publicly observable behavior, negative automatic thoughts, and inferred underlying cognitive structures or schemas [15,16]. When applying CBT to older adults with depression, several modifications should be made [17].

We developed two manuals: a training manual and a therapist manual. The training manual was used in the training sessions. The training manual provided information about mental disorders and geriatric depression, as well as basic counseling skills and cognitive behavioral therapy. The therapist manual was developed according to the degree of understanding and acceptance of village doctors and older adults in the rural Chinese context. The therapist manual provided a step-by-step guide for each session. A typical session contained the following steps: setting the agenda, a physical examination, identifying emotions, psychological interventions, and providing a “prescription”. The psychological intervention included cognitive and behavioral strategies that could be mastered and accepted by both the VDs and the rural elders. Cognitive strategies included identifying stressful situations, negative mood states, negative automatic thoughts (NATs), as well as identifying the advantages and disadvantages of NATs and examining evidence for and against NATs. Behavioral strategies included monitoring and scheduling activities, teaching problem solving skills, and practicing relaxation exercises. Homework was referred to as the “prescription”, which was more acceptable by the rural adults.

#### Training, intervention and supervision

VDs received six full days (three consecutive weekends) of training. The first weekend training included a one-day workshop that presented information on mental disorders such as depression, anxiety disorders, bipolar disorder and schizophrenia, and one-day workshop that introduced basic counseling concepts and techniques such as active listening, paraphrasing, summarizing, and empathizing. The second-weekend training focused on geriatric depression: its symptoms, causes, and treatments (the third day), and cognitive behavioral therapy: its history, theory and techniques (the fourth day). The third-weekend training focused on teaching VDs to implement BC-CBT across eight therapy sessions. Role play was used to practice skills, while small-group and large-group discussions were held throughout the entire training. The trainings were conducted by one psychiatrist, one licensed psychologist, and one qualified cognitive therapist trained at the Beck Institute in the USA. After the training, the VDs contacted the elders and initiated the two-month intervention, which involved eight weekly sessions, an average of 40 minutes each, in either the participants’ home or the clinic.

All VDs were monitored and supervised by the licensed psychologist throughout the entire intervention. To encourage fidelity to the manuals, every session was recorded for review. Supervision sessions typically included (a) feedback to VDs based on the review of intervention sessions, (b) discussion about the difficulties and sharing feelings about the past week’s intervention, and (c) didactic discussion of the case presented in supervision by the supervisee.

### Participants

The qualitative study was conducted in ten villages from October 2014 to January 2015 in *Mianzhu*, *Sichuan* province, China. Mianzhu has relatively more affluent rural areas. In 2014, the per capita net income of rural residents of this city was 12,501 yuan (approximately 2,014 US dollars). Meanwhile, the per capita net income for China's rural residents was 9,892 yuan (approximately 1,594 US dollars).

Ten village doctors voluntarily participated in the training workshops and supervision, and completed the intervention with a total of 19 older adults. None of these VDs had any previous training in psychotherapy. Criteria for participation in the intervention for older adults included: a) Center for Epidemiologic Studies Depression Scale (CES-D) score ≥ 16, 2) GDS score ≥ 10 and 3) Mini-mental State Examination (MMSE) score ≥ 18; and d) over 60 years old. Older adults who had been diagnosed with cancer, dementia, and severe mental illness were excluded. Informed consent was obtained from all participants meeting the criteria. Literate participants signed the consent form themselves. Illiterate participants signed their names with the aid of researchers.

### Data Collection

We conducted one focus group discussion with a total of 10 village doctors. Individual interviews with each village doctor and each older adult were held in their private residences. The focus group discussion (FGD) with the VDs was carried out at a private meeting room of Mianzhu Maternal and Child Health Hospital, while the individual interviews (IIs) with VDs and elders were held in private residences based on the convenience of the participants. These locations helped maintain privacy and confidentiality. The subjects were encouraged to share their experiences freely. Care was taken to see that all the participants contributed to the discussions. The FGD and IIs were conducted immediately after the completion of the intervention and supervision.

The village doctors’ topic guides (see Table 1) inquired in great detail about the doctors’ experience of the entire intervention program including the training, intervention, and supervision sessions. They were asked to identify the advantages and challenges in these sessions. The topic guides (see Table 2) developed for the older adults explored their experiences of the intervention. We also investigated the barriers and motivating factors to the intervention program, and their willingness to employ the treatment when the program ended.

**Table 1:** Focus group discussion questions and individual interviews with village doctors.

**Advantages**
How have you found the experience of taking part in the intervention program?
What are the potential positive results of taking part in the program?
How did you find the training sessions?
What are some of the factors in the training that make you capable to conduct the intervention?
How do you find the intervention sessions?
What are some of the factors during the intervention that make the intervention work?
How do you find the supervision sessions?
What are some of the factors during the supervision that improve your ability?
What would help to keep it going?
Do you think it would be feasible to add this into your normal practice?
**Challenges**
What were the challenges in the overall intervention program?
What are some of the factors in the training that make it difficult for you to master the CBT theory and techniques?
What are some of the factors in the intervention that make it difficult for you to continue your service?
What are some of the factors in supervision that make it difficult for you to improve your ability?

**Table 2:** Individual interviews with older adults.

**Advantages**
Was the intervention helpful for you?
What are some of the factors in the intervention that make it helpful for you?
Was there anything (knowledge or skills) you will keep using?
Are you willing to receive the intervention if the doctors continue talking with you after 8 sessions?
**Challenges**
What were the problems you came up against when receiving the intervention?

All interviews (including FGD and IIs) were conducted by the same researcher (XT). Field notes were taken during the interviews. The FGD lasted approximately three hours. The IIs for village doctors lasted 30 to 50 minutes, while the IIs for older adults lasted 10 to 20 minutes. Saturation was reached at the end of data collection with the emergence of consistent themes; however, interviews continued to ensure that all participants who took part in the program had their voices included.

### Data Analysis

All interviews were conducted in the Sichuan dialect and audio-recorded. The recordings were transcribed verbatim into Mandarin by an experienced local transcriber. The transcripts were then analyzed using thematic analysis [18]. Following the initial coding, in which labels were attached to text segments indicating important material in relation to the research questions, analysis continued with repeated reading of the transcripts to develop a set of themes that captured the essence of the FGDs and IIs. Two researchers (XT and FY) coded each transcription, and identified categories and themes separately. Discrepancies were resolved by discussion between the two researchers, and a thematic framework was developed and agreed on by consensus. To improve the validity and reliability of the analysis, the research team regularly met to check and discuss the emerging themes and issues. Coding and categorizing was carried out using NVivo 8.

## Results

### Practitioner characteristics

All 10 village doctors were of Han Chinese ethnicity and aged 36 to 44 years (mean age 40.8 years). The VDs were predominantly female (2 males, 8 females) with an average duration of practice of 16.7 years. All of the subjects except one were married. The majority (8/10) of them graduated from technical secondary schools. One had junior college education and one had senior high school education. All doctors had obtained a Village Doctor Certification. Three passed the National Licensed (Assistant) Doctors Examination, and two of them became licensed assistant doctors while the other one became a licensed doctor. During the trial, they treated on average two patients, who attended on average eight sessions.

### Service user characteristics

Village doctors provided intervention for older adults who had a score of 9 or more on the Geriatric Depression Scale (GDS) and 15 or more on Center for Epidemiologic Studies Depression Scale (CES-D). The mean age of the 19 elders was 73 years (Min = 64, Max = 90, SD = 6.94) and 12 (63%) were male. Only three (15.7%) had above a middle school education; six (31.5%) were divorced or widowed; all but one had at least one chronic disease.

The findings of the themes and subthemes from the qualitative analysis are summarized below and are accompanied by quotations that illustrate key points. Advantages and challenges results are reported separately. Table 3 and Table 4 elaborates the themes and the key findings.

**Table 3:** Themes and Key Findings from VDs and older adults: Advantages.

Themes	Key findings
Intervention Program Setting	Training
	• Knowledge concerning mental disorders, psychological counseling, geriatric depression and cognitive behavioral therapy
	• Role play
	• Large numbers of illustrative examples
	• Using instructive manuals and guides
	• Three consecutive weekends of training
	Supervision
	• Learning from others’ cases and receiving direction for working on their own cases
	• Held every weekend to monitor the intervention
	• Writing case reports
	• Discussing difficulties and sharing feelings
Delivering agent	• Strong interest
	• Strong ability to learn
	• Having previous knowledge about mental health issues
	• Being a local resident
	• Multiple roles: doctors, public health providers, public service providers
Favorable results for doctors	• Helping address cognitive, emotional and behavioral problems in themselves and helping them better communicate with their family and patients
	• Financial incentives
Service users’ needs	• Feeling lonely and depressed
	• Sharing private matters
Favorable results for elders	• Receiving physical exams and receiving information and advice concerning physical health
	• Learning skills to improve mood and interpersonal relationships
Cultural and political factors	• Renqing in Chinese context
	• Perceived as a government policy consideration

**Table 4:** Themes and Key Findings from VDs and older adults: Challenges.

Themes	Key findings
Challenges for the Intervention Program	Training
	• Lack of demonstration of the real treatment process, which is considered more powerful than the use of simulated patients and role-play
	• Lack of step-by-step guides for interventions for specific problems of depressive older adults
Challenges for doctors	• Time constraints and busy workloads
	• Anxiety and loss of confidence
	• Poor adherence to the protocol
	• Feeling incompetent in implementing the intervention
Challenges due to the elders’ family	• The presence of others (spouse, sons or daughter)
Challenges for the elders	• Those who are in good condition have no time to receive intervention because they have to work
	• Suffering from serious problems (physical, financial, et al.), which lead them to believe the intervention will be ineffective

### Advantages

Both village doctors and the older adults identified several key advantages of implementing the intervention for geriatric depression in Chinese rural areas. Six main themes emerged: the intervention program setting, the delivering agent, favorable results for the doctors, service users’ needs, favorable results for the elders, and cultural and political factors. As the findings included the interviews of both village doctors and older adults, for the sake of clarity, the subjects in the results presented below are village doctors, unless explicitly stated otherwise.

#### Intervention Program Setting

**Knowledge of mental disorders, psychological counseling, geriatric depression and cognitive behavioral therapy:** The majority (9/10) described the knowledge mentioned above as useful and easy to understand. The knowledge about mental disorders helped the doctors to distinguish depression from other disorders such as anxiety. It also provided the basis for learning and implementing CBT techniques.

*If we do not know the theory and basic information about mental disorders or cognitive behavioral therapy*, *we cannot master the skills in the following training sessions*. *And this knowledge helps us to identify what the client suffering from*, *depression*, *anxiety or mania*.

**Role play:** All VDs highlighted the importance of role-playing in the training sessions, describing it as particularly useful. It was more effective in helping VDs retain knowledge and develop skills than just listening or reading. The therapeutic environment seemed real and also led to more engaging interaction by learners.

*Role play is of great use*. *It seemed that I had mastered the skills by listening*, *reading and watching*, *however I realized how inexperienced I was when I conducted the role play*. *The role play improves my counseling skills*.

**Large numbers of illustrative examples:** Eight out of 10 VDs reported the value of the large numbers of illustrative examples used in the training sessions. Most illustrative examples, which were carefully designed, enhanced the presentation of materials throughout the training, and aided the understanding of the theory and practice of the intervention.

*Those examples are very helpful*. *They vividly illustrated the theory and techniques of the intervention*. *And they originate from everyday life so we can easily understand*.

**Using instructive manuals and guides:** Manual and guides were viewed positively by all, being described as indispensable tools to complete the training and deliver the service. The manuals and guides assisted VDs to quickly grasp essential knowledge and review what they had learned during the training. In addition, VDs usually looked over the manuals before they started a counseling session.

*These guides save me time because I do not have to take notes all the time*. *And they provide me an overview of the key components of the intervention*. *Before I go to the client’s home to conduct a session*, *I usually take a look at the intervention manual*, *which reminds me of techniques and important information*. *They are necessary for beginners*, *including me*.

**Three consecutive weekends of training:** Over half (6/10) spoke positively about the arrangement of three consecutive weekends of training, which made it possible for the VDs to develop skills in a relatively short time. The intervals between two weekends provided enough time to review the knowledge and techniques. In addition, VDs easily forgot what they had learned if the intervals were too long. The rest of the VDs, however, expressed that it was rather difficult for them to take three consecutive weekends off as they had a number of patients to treat and other obligations.

*The training arrangement is appropriate*. *If the intervals are too long*, *I would easily forget what I have learned*. *Another disadvantage of prolonged intervals*, *I think*, *is we may procrastinate studying and practicing*.

**Learning from others’ cases and receiving direction for their own cases:** All 10 doctors stressed the importance of supervision, which provided an opportunity to learn from others’ cases and direction for their own cases. From the discussion of the cases, village doctors learned solutions to various problems and garnered insights from the other therapists and their supervisors. The supervision also provided professional suggestions and strategies to enhance their ability to cope with difficult cases.

*Views of a single person may be incomplete*. *Others’ opinions and suggestions can make up for these limitations*. *Sometimes I could not find a solution to a certain problem*, *and others came up with some good ideas*.

**Supervision held every weekend to monitor the intervention:** Half of the VDs reflected that the weekend supervision had an important role to play in monitoring the ongoing intervention. It ensured that the VDs conducted at least one session per week, regardless of their busy schedule.

*If there were no weekly supervisions*, *I might have postponed the intervention when I had other urgencies*. *However*, *the supervisions every weekend forced me to abandon the idea that I could conduct the session the following week because I did not want to be criticized by the supervisor*.

**Writing case reports:** All gave positive feedback in the writing of case reports shared in the supervision session. The benefits of the case reports included the following: (1) obtaining an overview of the present case, (2) recognizing inappropriate words that might be harmful to the intervention or the client, and (3) brainstorming more appropriate solutions.

*When I was writing a case report*, *I found some words were inappropriate*, *which harm to the client*. *And sometimes I did not know how to address a situation when I was conducting the counseling*. *Writing the case report gave me inspiration*.

**Discussing difficulties and sharing feelings:** Almost all of the VDs (9/10) felt that supervision provided a safe environment to discuss difficulties and share feelings. In addition to the function of improving counseling ability as mentioned above, supervision also provided emotional support to the practitioners.

*It is important to discuss the difficulties with the supervisors*. *Other doctors and the supervisors usually provide excellent advice*. *I often heard other people confront the same problems I had*. *Some were even more worried and anxious than I was*! *I realized that I was not alone*. *The supervisor often told us*, *“It is rather tough to work with ‘rural’ ‘depressive’ ‘elders’*. *Even an experienced counselor may not handle the three aspects well*.*” I felt relieved when I heard this*.

#### Delivering agent

**Strong interest:** All the VDs referred to their interest as particularly important and decisive. The doctors were interested in the program because of the benefits the program offered. The theory and techniques they learned were beneficial to themselves, their families, and their patients. The various benefits will be presented later in the paper under another subheading.

*Interest was vital for me to participate in the program*. *If a person did not have interest*, *he/she cannot benefit from the program*. *I was interested in the program because of the following reasons*: *Firstly*, *I wanted to enlarge my scope of knowledge*, *which I believe was quite narrow; secondly*, *I had an uncle who suffered from depression many years ago*, *and a cousin who had a depressive episode during pregnancy*. *I realized depression is quite common and I wanted to know how to address it; thirdly*, *a number of patients come to my clinic with certain diseases*, *which come with related psychological issues*. *I hope I can use what I have learned from the program*, *combined with medicine*, *to help them*.

**Strong learning ability:** All of the doctors spoke of their strong learning ability in mastering a new therapy. Most of the village doctors graduated from technical secondary schools. More importantly, they are continuously learning in their field, including attending trainings held by the Health Bureau or other official institutions, and pursuing qualification certificates as licensed doctors.

*Well*, *I do not think I am a person of high intelligence*. *Compared with ordinary villagers*, *maybe I have a slightly better learning capability*. *After all I have been preparing for the National Licensed (Assistant) Doctors Examination*, *and I have many books to read*.

**Having previous knowledge about mental health issues:** All of the VDs recognized their previous knowledge regarding mental health issues had a significant benefit in their participation in the program. Previous schooling had provided some education in mental health. Additionally, the doctors had received training in mental health since their careers in medicine began. This knowledge made the intervention program much easier for them to accept.

*I had some knowledge about this (mental health)*, *and then I came to your program*. *I felt it was not very difficult for me to understand*. *At least I heard of depression*, *psychological counseling*, *and the other concepts before*.

**Local residents:** Most of the VDs (8/10) mentioned that being a local resident aided in the intervention. All older adults who received the intervention expressed that they trusted local village doctors. In a village with two or more VDs, a VD may find it difficult to conduct interventions with the elders in the area who are not his or her own patients.

*There are advantages of being a local resident*. *They all know me*, *and some clients are even my distant relatives*. *They trust me and allow me to talk with them*. *I cannot imagine a stranger showing up in their house and discussing very private issues*. *They might regard the stranger as a charlatan or someone spreading a heresy*. *(Village doctor)*

*She is local*, *and I go to her clinic when I am sick*. *I know her very well*. *Of course I trust her*. *(Older adult)*

**Multiple roles: doctors, public health providers, public service providers:** The multiple roles of the therapists participating in the intervention were highlighted as particularly significant by the majority of respondents (9/10). The therapists were village doctors who provided primary health care; public health providers who provided public health services to rural residents with diabetes and hypertension; public service providers who sometimes helped higher level health departments carry out work. These roles helped foster a strong relationship between the village doctors and the local older adults.

*I am a village doctor*. *I treat them and help them manage their diseases*. *They trust their doctor*. *And they tend to believe that a doctor has adequate professional knowledge*. *In these years we have been providing public health service for the elders*, *it has been free*. *They feel we are concerned about them and thus develop a close relationship with us*.

#### Favorable results for doctors

**The program helps the doctors address cognitive, emotional and behavioral problems in themselves and better communicate with their family and patients:** All of the village doctors highlighted the value of the intervention program, which helped them address their own cognitive, emotional and behavioral problems and the relationships with their family and patients.

*We often face various troubles in our everyday life*. *After I learned this intervention*, *I used it to treat myself and my family*. *Last year something terrible happened in my family*. *It was a really tough time*. *The whole family was haunted by negative moods and obsessed with catastrophic thinking*. *Therefore*, *I used what I had learned to help myself*, *my parents and other family members*. *In my clinic*, *some diseases are accompanied by psychological factors*. *Therapeutic techniques*, *combined with medicine*, *are more effective than treating them by merely taking medicine*.

**Financial incentives:** The majority (9/10) indicated that financial incentives were important and necessary. Village doctors have no fixed monthly salary. Their income is mainly decided by the amount of medicine they sell. The intervention took time from their earning capacity. Further, if patients came to the clinic several times but did not see the doctors they knew, they may turn to other doctors, resulting in the loss of a potential patient. Therefore the subsidies provided by the intervention program seemed very important for them.

*The subsidy is important for us*. *It motivated us to continue the delivery of the intervention*. *We do not have salaries*. *We depend on the income of selling medicine*. *We have an additional public health subsidy from the government*. *The intervention session for one older adult per week takes me one hour*. *And I have to spend some time on the road*. *If the patient is not at home*, *I have to come again*. *Therefore a subsidy helps increase our enthusiasm for the intervention*.

#### Service users’ needs

**Feeling lonely and depressed:** In interviews with older adults, 13 out of the 19 respondents shared that they felt lonely or depressed and that if they had someone to talk with, they imagined that this would be improved. A portion of participants lived alone or were divorced or widowed. A portion of participants lived with their partners, who suffered from severe or multiple disabilities and could not communicate well, leaving them with few people with whom they could socialize.

*My son died a few years ago*. *I have been in sorrow since*. *I do not talk with my partner about the death of our son*. *It is a really sad thing for both of us*. *However*, *I miss him so much*.

**Sharing private matters:** A portion of older adults (5/19) reported that they could share private matters with the VDs, including emotional problems caused by interpersonal factors, such as relationships with adult children or daughter-in-laws. Normally, these types of private family matters are not discussed openly. They could share these burdens with their therapists in a safe environment.

*You know*, *it is an ill bird that fouls its own nest*. *No one would share these things with others*. *If my family members knew*, *they would definitely quarrel with me*.

#### Favorable results for elders

**Receiving physical exams and the provision of information and advice concerning physical health:** All older adults reported that they had received physical examinations. The village doctors performed a routine physical exam at the beginning of the intervention session, which included measuring blood sugar levels, blood pressure and so on. They offered their clients a basic overview of their physical conditions. The doctors also provided relevant advice and information to the patients, including suggestions about daily exercise, diet, sleep, and so on. The clients may also have received further suggestions to go the hospital and obtain a complete physical examination.

*Every time [I visit the doctor] the doctor measures my blood pressure as I have high blood pressure*. *The doctor is very kind to me*. *She reminds me to eat a healthy diet and get regular exercises again and again*. *I go to her clinic to buy some medicine and now I am starting to pay attention to my blood pressure*.

**Learning how to improve mood and interpersonal relationships:** More than half (10/19) mentioned that they had learned skills to improve mood and interpersonal relationships. Most of them adopted some behavioral strategies, such as walking, talking with others, or playing cards, to increase positive emotions. Others used cognitive strategies—like seeking evidence for and against one’s partner’s negligence, or identifying the advantages and disadvantages of regarding ones daughter-in-law as a bad person—to engender cognitive and emotional changes.

*The doctor told me to think about my wife’s good and bad sides*. *I rejected the idea of thinking of my wife’s merits at first*. *The doctor*, *however*, *suggested it as homework which I must complete*. *It took me some time to think of those*. *I wrote them down*. *And I found that she actually had so many merits I had ignored for a long time*.

#### Cultural and political factors

***Renqing* in Chinese context:** Four out of ten village doctors discussed one reason why older adults may have been willing to receive our intervention: *Renqing* is a set of social norms by which one abides by to get along well with other people in Chinese society [19]. Most of the older adults (15/19) also reported that it was impolite and rude to reject the doctor’s kindness. The interpersonal relationships are quite close in Chinese rural areas. This factor created a favorable condition for the successful implementation of the intervention program.

*Maybe the patient did not have the heart to refuse my intervention*. *Sometimes I felt that*. *Sometimes everything is going well for him*, *and he still accepted my intervention*. *(Village doctor)*

*The doctor came to my house to chat with me*. *She spent large amounts of time and asks for nothing*. *How can I reject her*? *It is very rude and inappropriate to reject her kindness*. *(Older adult)*

**Perceived policy consideration:** The majority of the village doctors (9/10) noted that the elders believed the intervention program was a government service, similar to the public health services provided for them, which was always supported by the elders. More than half of the elders (10/19) referred to the intervention program as a government policy. They appreciated and were grateful for the government’s efforts. Sometimes they even thought our investigators were sent by the government to collect their information for further use.

*Many elders think it is a government policy*, *which they highly praised*. *They often say to me*, *“the government is so kind to the elders”*. *Thus our intervention continued smoothly*. *(Village doctor)*

*The government is so kind*, *and so is the communist party*. *You are so kind to our old people*! *(Older adult)*

### Challenges

Four primary themes emerged from the thematic analysis: (1) challenges for the intervention program, (2) challenges for the doctors, (3) challenges from the elders’ family and (4) challenges from the elders. These themes enabled us to identify factors which impacted whether the intervention program was likely to work well, and where it was likely to be more problematic. The themes and subthemes are discussed below.

#### Challenges from the intervention program

**Lack of demonstration of a real treatment process, which is more powerful than the use of simulated patients and role-play:** The majority (8/10) suggested that treating a real patient in the training would be more impressive and helpful to them. They said they could feel the effectiveness of the cognitive behavioral therapy through conducting a counseling session with a real (depressive) patient. Additionally, role-playing is based on imaginary problems. Sometimes people could not go deeper because they were not able to come up with an underlying reason in a state of nervous tension during the role-play.

*Treating a client with real problems may be better*. *One time the trainer conducted a real counseling session with Doctor Tian*. *Tian told the therapist her real troubles*, *not imaginary ones*. *We were deeply impressed by the process of intervention*. *It was awesome*! *And we realized that there were a lot more things we need to learn*. *In a real counseling session*, *we observed the flexible methods the therapist used to address various situations*.

**Lack of step-by-step guides for interventions based on the variety of problems of depressive older adults:** Most of the doctors (7/10) agreed that it would be better if the manual incorporated step-by-step guides for interventions based on the problems of the rural depressive elders. The manual did not specify that the VDs should strictly obey the structured manual. Instead, it provided various possible methods for beginners to use in conducting a session.

*It would definitely be better for us beginners*. *It would reduce the difficulty of conducting a session*. *However*, *the former manual was good enough*. *It may be lack of capacity that resulted in a lack of skillfulness during the delivery*. *If the manual included steps based on various physical or economic problems or relationships with the family*, *it would be perfect*!

#### Challenges from doctors

**Time constraints and busy workloads:** Six out of 10 VDs found that time constraints and busy workloads made them feel stressed. VDs usually had a number of tasks to do: treat patients, conduct public health services, farm, and take care of their children, and so on. They had to find ways to maintain balance in all aspects of life. Three people mentioned the weekly supervisions were stressful because they had to squeeze time out of their hectic schedules. All VDs, though busy, attended the supervisions with no more than one absence.

*If there are many public health services to provide in that period of time*, *it may conflict*. *In addition*, *those who had fewer family members had to pick up their children on Friday afternoon and take them to school on Sunday afternoon*.

**Anxiety and loss of confidence:** Many VDs (7/10) felt anxious before they conducted a session, and lost confidence when clients dropped out of session. Their anxiety was affected by the outcomes of their patients. If the patients gave positive feedback, they felt less anxiety the subsequent visit. All seven VDs expressed that supervision had offered advice and support to help them feel relaxed and regain their confidence.

*Sometimes before I conducted a session*, *I felt anxious and even doubted myself*. *Can I really help him overcome his troubles*? *At the end of the session I asked for feedback from the patients*. *If she/he feels good*, *I relaxed*. *If there was not any improvement*, *I felt sad and more anxious*. *It would be hard to move on*. *Therefore*, *I hoped the supervisor would help me*.

**Poor adherence to the protocol:** Four out of 10 doctors found there were times they could not adhere to the protocol. The doctors analyzed the reasons that might contribute to this: firstly, they “knew” theoretically how to conduct a session step-by-step. When it came to implementation, the people could not “perform” it well. Secondly, there were numerous factors that interfered with the sessions, such as the presence of others and uncooperative patients.

*Sometimes I felt unskillful in using the techniques*. *I was supposed to respond in this way at that time*, *however I failed*. *Sometimes during the intervention*, *the grandson came home from school*, *or other people dropped by*, *and my client would talk with them*. *It was hard to do as the manual told me in these situations*.

**Feeling incompetent in implementing the intervention:** Half of the VDs felt incompetent in implementing the intervention. This might partially result from the poor adherence to the protocol. Lack of communication skills might have also contributed to the doctors’ perceived incompetence.

*The manual told me to chat with the patient about any topic when entering the patient’s home*. *However*, *I usually did not know what to talk about*. *It really embarrassed me*. *I am wondering whether counseling is a suitable work for me*.

#### Challenges for the elders and their family

**The presence of others (spouse, sons or daughter):** All VDs expressed that one of the biggest challenges of the successful implementation of the program was the presence of others. It had the following two negative effects: (1) elders could not talk freely, especially when the problems resulted from being mistreated or neglected by the family. (2) Others might interrupt the process of intervention.

*One of my patients sold his farm-made agricultural products at a market during the day*. *I had to talk with him after supper in his house*. *His son and daughter-in-law lived with him*. *My patient had some ideas that were inconsistent with his son*. *He mentioned this point every session*, *but never went deeper*. *He worried about being heard by his son*.

*When I came up with an idea about the steps I would take to treat the patient*, *his neighbor*, *standing beside him all the time*, *spoke loudly*, *trying to dissuade my patient from thinking in this way*. *I had to turn to him*, *telling him to keep silent*. *After that incident*, *I forgot what I should do with my patient*.

**Those who are in good condition have no time to receive the intervention because they have to work:** Three out of 19 older adults found it stressful to squeeze time out of their busy work. The three respondents were relatively healthier than the other elders. Although they have passed the retirement age, they still worked in the factories near the village or sold agricultural products in the markets. The VDs had to negotiate with in arranging the sessions.

*I am too busy during the day*. *My wife is in poor health*. *I have to get up early in the morning to arrive at the market so that I can secure a place to sell my products*. *And I do not come back home until I sell out all the goods*. *It is often very late*.

**Some older adults suffer from serious problems (physical, financial, et al.), which lead them to believe the intervention will be ineffective:** Several elders (5/18) said serious physical problems or other issues impeded the process of intervention. Physical problems included serious sleep problems, severe pain, and persistent numbness. Other issues included financial trouble, and the inability to obtain basic living allowances or other subsidies. People with these problems tended to view the intervention as useless.

*I do not think the intervention would work on me because the doctor cannot help me get basic living allowances*. *He does not work on the village committee*.

## Discussion

### Principal findings

The findings of this qualitative study provide useful insights into the nature of training, intervention, and supervision, as well as other favorable factors resulting from the delivery of the program. The village doctors stressed the importance of role-playing and instructive manuals in the training. Receiving information about mental disorders, CBT, and illustrative examples were also viewed positively by most of the doctors. Supervision was a vital part of the program. VDs learned from others’ cases and received direction for their own. The doctors also benefited from writing case presentations and obtained emotional supports in the supervision. Our findings highlight the role that village doctors may be able to play in providing psychological interventions for depressive elders. Their interest, learning capability, knowledge about mental health issues, and multiple roles make them highly suitable for delivering the intervention in rural areas. *Renqing* between older adults and VDs and the perceived governmental involvement made this intervention more accessible to these depressive elders.

This study also highlighted a number of potential difficulties and barriers to the successful implementation of the intervention. Lack of real therapy in the training and step-by-step manuals impeded the development of competency. Challenges also arose for the village doctors, including time constraints, anxiety, feelings of incompetence and poor adherence. Other barriers included the presence of other people when conducting the intervention, and sometimes the inability to find clients in some older adults who were in good health had no time to receive treatment.

CBT is one of few psychological interventions that have been found to be effective for depression in low-income and middle-income countries [20–22]. CBT has also been proved to be effective for reducing depression in older people [7]. However, the use of CBT by lay health workers with little understanding of psychotherapy for depression in rural elders has not been tested before in a developing country. A number of people think that it is too difficult for lay health workers to master this relatively sophisticated technique. Additionally, older adults prefer to take medicine rather than receive CBT [23]. However, closer examination of the setting and population reveal that there are numbers of factors that may facilitate the successful delivery of such an intervention.

### The importance of context

People with mental illness may encounter public stigma and suffer from self-stigma [24]. This stigma may occur more frequently in China, as it may lead to loss of moral status and social capital [25]. Thus, although many elders suffer from depression, few of them are willing to seek treatment from mental health professionals [26]. However, village doctors have several advantages that may assist them in providing services to the elders. First, doctors live and work in the same village as patients. They may be neighbors, relatives or friends of the older adults. Thus doctors and patients knew each other prior to the intervention program. Since 2009, village doctors have been required to provide both medical and public health services to the rural residents. They maintain health records for all the rural citizens and conduct regular examinations for rural residents aged 65 or older with diabetes and hypertension. All of these factors enhance familiarity and build trust between doctors and patients. As Chinese people may be less willing than their Western counterparts to report psychological problems during face-to-face intervention with unfamiliar mental health professionals, the familiarity and trust between patients and doctors is important for the intervention [27]. Second, the majority of the VDs aged 50 or younger have graduated from technical secondary schools or above [28]. The on-the-job training and ongoing pursuit of National Licensed (Assistant) Doctors keeps them in a perpetual learning mode, which makes it easier for them to learn new techniques. They demonstrated interest, curiosity and enthusiasm in the program because the BC-CBT is helpful for their family, patients and themselves. These factors contribute to the village doctors’ suitability in delivering such an intervention.

*Renqing* is a unique term in Chinese culture. *Renqing* refers to a set of social norms by which one has to abide to get along well with other people. According to Hwang [19], this norm of *renqing* includes two basic types of social behavior: (1) Ordinarily, one should keep in contact with the acquaintances in one's social network, exchanging gifts, greetings, or visitations with them from time to time, and (2) when a member of one's reticulum gets into trouble or faces a difficult situation, one should sympathize, offer help, and "do a *renqing*" for that person. When the village doctors provide intervention to them out of kindness, older adults believe it breaks the norms of *renqing* and may offend the doctors if they reject the intervention. In the context of *renqing*, the elders are not likely to refuse the intervention. This unique interpersonal relationship in Chinese culture facilitates elders’ access to mental health services, which is normally one of the key barriers experienced by rural older adults [29].

### Training and supervisory needs

Like many implementation studies, experienced therapists provide short-term tailored training and supervision for lay health workers [30]. The village doctors in the present study stressed the importance of training and supervision. Role-playing and the use of instructive manuals and guides in the training were highlighted as particularly useful, which is consistent with other similar qualitative studies that examine the feasibility of implementing CBT [31–33]. As all the village doctors had little prior knowledge of counseling techniques, it was necessary to train them in mental health issues and the basic principles of psychological counseling, as well as CBT theory and techniques.

The feedback from the village doctors supports the importance of supervision in training lay health workers in CBT. A growing body of research has demonstrated therapist adherence and competence may be associated with effective supervision [34–36]. In this study, weekly supervision sessions for groups of 10 village doctors were carried out by a licensed psychologist. The supervisor provided feedback to village doctors based on the review of audiotaped sessions to promote adherence to the intervention protocol. Discussion of the case presentation and difficult situations helped maintain skills and enhance therapist competence. Sharing feelings of anxiety and the acknowledgment of the difficulty in treating rural depressive elders in the group helped the VDs relax and increased their confidence.

### Ideas for improvements

#### Step-by-step treatment manuals for specific problems

By analyzing the audiotaped therapy sessions of the 19 older adults, we identified the most frequently cited problems of rural elderly people. A more detailed step-by-step treatment for all types of problems should be developed and provided to village doctors. The revised manual can help the beginners develop skills and improve therapist adherence and competence.

#### Incorporating support from family

Incorporating possible external support (such as family members) may be beneficial to the intervention. First, the family can help facilitate a therapeutic environment if they fully understand and support the intervention program. More importantly, because Chinese adaptive coping responses are believed to occur at multiple levels of social organization rather than at the level of the individual, therapists need to consider the influences from multiple levels of social organization [37]. Incorporating the family members may facilitate client change in therapy and maintain therapeutic efficacy.

#### Integration into public health services

Since 2009, the Ministry of Health (MOH) has required local government agencies to subsidize village doctors in providing public health services to the rural residents [38,39]. These public health services include maintaining health records and conducting regular examinations for rural residents aged 65 or older with diabetes and hypertension. If the treatment for depression is integrated into public health services, village doctors can receive financial support from the government, which may increase their willingness to continue the intervention when our program ends.

## Limitations

This study has several limitations. The primary limitation is a potential sampling bias, as more motivated village doctors may have agreed to take part. Views from those who are not interested in or cannot participate in this program should be incorporated. Therefore caution should be applied before generalizing the findings to all doctors. Secondly, only two male village doctors were recruited for this study. There are more male village doctors than female doctors in *Mianzhu*. The views from the male doctors may differ from the ones of female doctors due to the different family roles and other possible gender-related factors.

## Conclusions

Village doctors and older adults provided detailed feedback on the Brief Comprehensive Cognitive Behavioral Therapy. Training and supervision contributed to the development of skills and improved fidelity to the intervention. The benefits of the intervention for both of the village doctors and the elders lead them to be willing to implement/receive this intervention. The characteristics of village doctors and certain special elements of Chinese culture foster the implementation of the intervention. Despite some improvement needed in the future studies, the present study has demonstrated that it is potentially feasible to train lay health workers to provide psychological therapy, such as BC-CBT, to depressive elders in Chinese rural areas.
